# Gnarled-Trunk Evolutionary Model of Influenza A Virus Hemagglutinin

**DOI:** 10.1371/journal.pone.0025953

**Published:** 2011-10-10

**Authors:** Kimihito Ito, Manabu Igarashi, Yutaka Miyazaki, Teiji Murakami, Syaka Iida, Hiroshi Kida, Ayato Takada

**Affiliations:** 1 Hokkaido University Research Center for Zoonosis Control, Sapporo, Japan; 2 PRESTO, Japan Science and Technology Agency, Saitama, Japan; 3 Faculty of Liberal Arts and Sciences, Osaka University of Economics and Law, Yao, Japan; 4 Department of Disease Control, Graduate School of Veterinary Medicine, Hokkaido University, Sapporo, Japan; 5 OIE Reference Laboratory for Highly Pathogenic Avian Influenza, Sapporo, Japan; 6 SORST, Japan Science and Technology Agency, Saitama, Japan; 7 School of Veterinary Medicine, The University of Zambia, Lusaka, Zambia; British Columbia Centre for Excellence in HIV/AIDS, Canada

## Abstract

Human influenza A viruses undergo antigenic changes with gradual accumulation of amino acid substitutions on the hemagglutinin (HA) molecule. A strong antigenic mismatch between vaccine and epidemic strains often requires the replacement of influenza vaccines worldwide. To establish a practical model enabling us to predict the future direction of the influenza virus evolution, relative distances of amino acid sequences among past epidemic strains were analyzed by multidimensional scaling (MDS). We found that human influenza viruses have evolved along a gnarled evolutionary pathway with an approximately constant curvature in the MDS-constructed 3D space. The gnarled pathway indicated that evolution on the trunk favored multiple substitutions at the same amino acid positions on HA. The constant curvature was reasonably explained by assuming that the rate of amino acid substitutions varied from one position to another according to a gamma distribution. Furthermore, we utilized the estimated parameters of the gamma distribution to predict the amino acid substitutions on HA in subsequent years. Retrospective prediction tests for 12 years from 1997 to 2009 showed that 70% of actual amino acid substitutions were correctly predicted, and that 45% of predicted amino acid substitutions have been actually observed. Although it remains unsolved how to predict the exact timing of antigenic changes, the present results suggest that our model may have the potential to recognize emerging epidemic strains.

## Introduction

The hemagglutinin (HA) molecule of influenza A viruses is the prime target of antibodies that neutralize viral infectivity. The strong immune pressure against HA in the human population selects a new variant every 2–5 years [1]–[6]. Thus influenza A viruses undergo antigenic changes with gradual accumulation of amino acid substitutions on HA, and the antigenic change is one of the primary reasons why vaccination is not a perfect measure to control seasonal influenza. Accordingly, influenza vaccine often requires replacement to avoid antigenic mismatch between vaccine and epidemic strains [7]. The decision of vaccine replacement must be made several months before a minor strain become dominant strain [8]. Thus the prediction of antigenic change of influenza A virus [2], [9]–[15] has been one of the major public health goals.

Phylogenetic analyses of HA genes of human H3N2 viruses have revealed the presence of a long main trunk and short side branches in their evolutionary tree. The main trunk has grown continuously from a pandemic strain in 1968 to recent epidemic strains, and tips of each branch reached a dead end on the evolutionary pathway [13], [14], [16]–[18]. This ‘cactus-like’ phylogenetic tree indicates that the viruses on the side branches do not produce next epidemic strains, while the viruses near the main trunk do contribute to the production of both an epidemic strain and its next epidemic strain. Although two or more antigenically different strains were known to co-circulate in a single epidemic season [13], [19], [20], the single-trunk phylogenetic tree indicates the diversity of the HA amino acid sequences at any point in time is relatively limited. The reason why only one trunk exists has yet to be fully understood, but several theories have been proposed to explain this phenomenon [21]–[23].

The aims of the present studies are to establish a practical model enabling us to predict the evolutionary direction of the virus that causes future epidemics and to examine the accuracy of the prediction based on the model. First we analysed relative distances of amino acid sequences among past epidemic strains using a method called multidimensional scaling (MDS) [24]. We found that human influenza viruses have evolved along a gnarled evolutionary pathway with an approximately constant curvature in the MDS-constructed 3D space. The constant curvature was reasonably explained by assuming that the rate of amino acid substitutions varied from one position to another according to a gamma distribution. The estimated parameters of the gamma distribution allowed us to predict the amino acid substitutions on HA in subsequent years with reasonable accuracy, indicating the potential to select suitable vaccine strains for the subsequent epidemic seasons.

## Results

**Figure 1:**
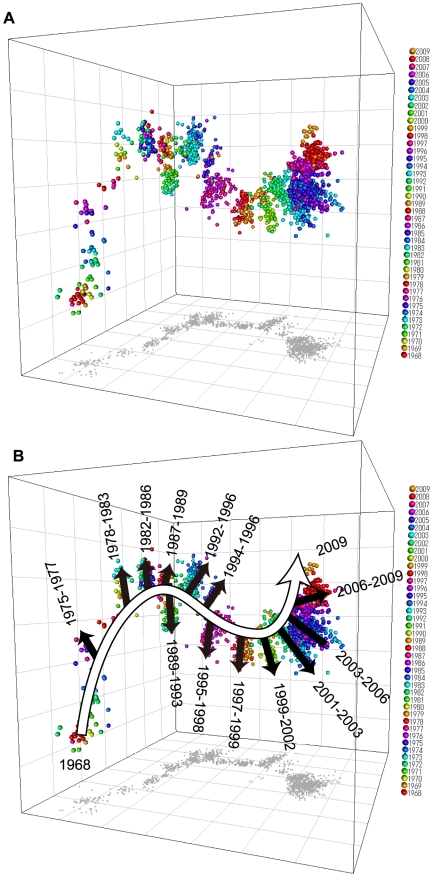
Three-dimensional map of HA sequences of H3N2 human influenza A viruses. A total of 2,640 amino acid sequences of the HA1 domain of human H3N2 influenza A viruses isolated during the period from 1968 through 2009 are visualized in a 3D space. Each point represents a virus strain. The distance between two viruses in the 3D map represents the number of different amino acids between their HA sequences. The whole coordination is determined by MDS analysis. The root-mean-square error of the 3D map was 1.72. All three axes represent the sequence dissimilarity (spacing between grid lines represents 10 different amino acids), and the configuration can be freely rotated and translated. Shadows represent projections of points onto the coordinate planes. (A) the 3D map colour-coded by the year of isolation of the virus. (B) a schematic diagram of the 3D map.

To expose underlying patterns of HA amino acid substitutions in the evolutionary pathway along the main trunk, we conducted multidimensional scaling (MDS) analysis [24] of HA sequences. The fundamental idea for visualizing a large number of sequences in a low dimensional space is based on the same idea described in a recent paper by He and Deem [15]. By performing MDS analysis, one may obtain a visual map of objects where the dissimilarity between objects is represented as the distance between corresponding points. A total of 2,640 unique amino acid sequences of the HA1 [3], [25] domain (328 amino acids long) of the H3N2 viruses isolated from humans during the period from 1968 through 2009 were analysed by MDS and visualized in a three-dimensional (3D) space (Figure 1, Movie S1). In the resulting 3D map, each HA sequence was represented as a point, and the number of different amino acids between two HA sequences was represented as the relative distance between two corresponding points. Although the original amino acid sequences provided 328 dimensional data, the numbers of different amino acids among sequences were reasonably approximated by distance in this 3-dimensional map with a root-mean-square error of 1.72 (Figure S1). Consistent with phylogenetic analyses, viruses that were isolated close in time were located near each other, forming a thick main trunk with short branches elongated from the trunk (Figure 1A, Figure 1B). The main trunk grew continuously from a pandemic strain in 1968 to recent epidemic strains. Each branch consisted of epidemic strains isolated during a period of 3–5 years.

It should be noted that the MDS representation revealed a characteristic feature that has not been clear only from phylogenetic trees, the observation of a gnarled trunk constantly curved in the 3D map. Since amino acid substitutions on human virus HA mainly occur in the HA1 domain [3], this result indicated that the evolution of H3N2 virus HA was characterized by this gnarled evolutionary pathway.

We also conducted the same analysis for H1N1 human viruses (Figure S2, Movie S2). The 3D representation of HA sequences of H1N1 seasonal influenza viruses showed the same pattern in their evolutionary pathways, a long gnarled trunk elongated from the pandemic strain in 1918. Another H1N1 pandemic strain, which was introduced into the human population in 2009 [26], was located at the end of a step-wise path that consisted of swine H1N1 influenza viruses isolated from humans from 1976 to 2007 [27].

**Figure 2:**
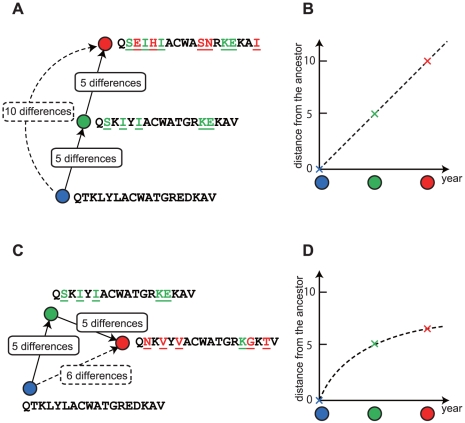
Two distinct patterns of amino acid substitutions that produce different spatial arrangements of viruses. (A) The straight arrangement of viruses. If a series of amino acid substitutions all occur in different positions, then the distance from an ancestor to a mutant should be proportional to the number of substitutions (B). These independent substitutions make a straight arrangement of viruses on the map. (C) The curved arrangement of viruses. If amino acids at particular positions were substituted more than once, the distance from an ancestor to a mutant should be less than the number of substitutions (D). These concentrated substitutions at the same amino acid positions make a curved arrangement of viruses. In both panels, viruses are represented by circles, with illustrative examples of their amino acid sequences and substitutions on them.

In the 3D map in Figure 1, the spatial distance between each pair of sequences represents the number of different amino acids between these sequences. Figure 2 illustrates two distinct patterns of amino acid substitutions that produce different spatial arrangements of viruses in this map. If a series of amino acid substitutions all occur in different positions, then the distance from an ancestor to a mutant should be proportional to the number of substitutions. These independent substitutions make a straight arrangement of viruses on the map (Figure 2A, 2B). On the other hand, if amino acids at particular positions were substituted more than once, the distance from an ancestor to a mutant should be less than the number of substitutions. These concentrated substitutions at the same amino acid positions make a curved arrangement of viruses (Figure 2C, 2D). For this reason, the gnarled trunk found in the MDS representation of HA sequences (Figure 1) indicated that HA variants on the trunk favoured multiple amino acid substitutions at the same positions. The fact that most of the amino acid substitutions occurred near antigenic domains A–E [3], [5], [9], [28] was consistent with the observation of the curved trunk in the 3D map.

**Figure 3:**
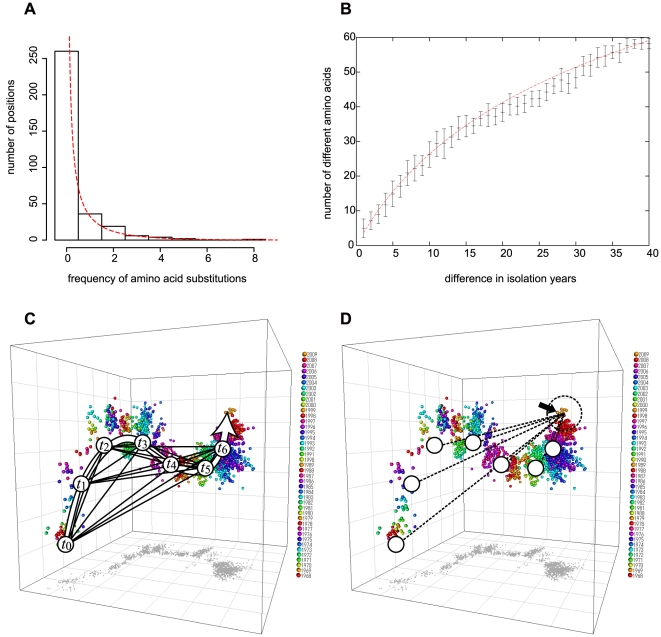
The amino acid substitutions on the gnarled trunk. (A) the distribution of the number of positions that were substituted on the trunk from 1968 to 2009. The gamma distribution that has a mean substitution frequency of 0.418 and a variance of 1.155 is superimposed. (B) The number of different amino acids between the trunk sequences plotted against the difference in their isolation years. The horizontal bars indicate mean values, and vertical lines indicate ±1 standard deviation of the number of different amino acids. A non-linear regression curve using the formula 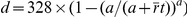 is superimposed (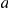 = 0.129, 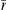 = 0.0118), showing good fit to the actual data (P<0.001). (C) A schematic illustration of trunk viruses and the sequence dissimilarities among them. A trunk virus is a virus located near the main trunk. Circles labelled with 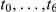 are examples of trunk viruses. The sequence dissimilarities among trunk sequences are shown by solid lines. (D) The selection of a Leading Bud. Using the formula of 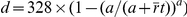, one may calculate the expected sequence dissimilarities between a future trunk virus and past trunk viruses (dotted lines). The bold arrow indicates a candidate for Leading Buds. For the coming influenza season in 2010, A/Thailand/CU-B110/2009(H3N2) was predicted to be the most likely candidate virus (Table S2).

To investigate the property of the curvature of the main trunk, we analysed the distribution of the number of positions that were substituted on the trunk from 1968 to 2009 (Figure 3A, Table S1). Of the 328 positions on the HA1 sequence, 260 remained unchanged for 41 years. At 36 amino acid positions, residues were substituted once, and at 19 positions twice. The number of positions gradually decreased as their observed frequency of substitutions increased, but there was one position that has substituted eight times. The mean of the substitution frequencies was 0.384, and the variance of the substitution frequencies was 0.904. Given these statistics for the substitution frequency, the shape of the histogram is almost identical to the curve of a gamma distribution [29] having the same mean and variance (Figure 3A). From this result, it is likely that the rate of amino acid substitutions varies from one position to another according to a gamma distribution.

To estimate the parameters of the gamma distribution precisely, non-linear regression analysis was performed. First, 91 HA sequences near the main trunk were selected as trunk sequences. In Figure 3B, the number of different amino acids between two trunk sequences (Figure 3C) is plotted against the difference in their isolation years. It is known that if the amino acid substitution rate varies according to a gamma distribution, the expected number of different amino acids between two sequences can be calculated by the formula: 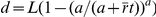, where 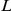
is the length of the sequences, *a* is the shape parameter of the gamma distribution (gamma parameter), 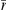 is the mean substitution rate, and 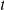 is the difference in the years of the two sequences [30]. By fitting the above formula to the actual numbers of different amino acids on the trunk, the gamma parameter and mean substitution rate were estimated (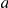 = 0.129 and 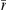 = 0.0118), showing a good fit to the actual data (P<0.001). This result indicated that the number of different amino acids between two sequences could be determined from the difference of the year of isolation, according to the gamma-distribution-based model presented above. Thus, it is reasonable to conclude that the constant curvature on the trunk in the MDS representation (Figure 1) was attributed to this nature.

Next, an attempt was made to apply the gamma-distribution-based substitution model of the trunk to the prediction of amino acid substitutions in subsequent years. The key idea of our prediction method is to select the direct progenitor virus for future epidemics from the surveillance samples of each year. We designate a virus strain that is located near the trunk extending into the next year as a Leading Bud. A Leading Bud can be considered as an potential dominant strain that is not dominant in the given year and become dominant the next year [15]. Using the formula 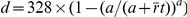
under the estimated 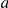
and 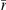, one may calculate the expected number of different amino acids between two HA sequences located on the trunk. According to this gamma-distribution-based formula, a virus that will appear on the trunk in a particular year is expected to have 4, 7, 10, 13, ... , 60 different amino acids in HA, when compared with viruses in 1, 2, 3, 4, ... , 42 years before, respectively. Therefore, given the large variety of viruses isolated in a year, the virus that is likely to be located near the extended trunk is the virus to which HA sequence dissimilarities from past viruses have the highest fit to those expected under the gamma-distribution-based formula (Figure 3D).

To examine whether the method correctly selected the Leading Bud in the subsequent year, we conducted retrospective tests for each year from 1997 to 2009. Evaluation was made by comparing the predicted Leading Bud with the dominant sequence of the subsequent year, which consisted of the amino acids that constituted the majority at each position in the year. Table 1 shows the results of retrospective tests (for details, see Table S2). The recall, which is the probability that an actual substitution was correctly predicted, was 1.00 in 4 of the 12 calendar years. The overall recall of the prediction was 0.70, indicating that the model had a reasonable ability to predict amino acid substitution in the subsequent year for each year. The precision, which is the probability that a predicted substitution actually occurred, varied from 0.0 to 0.89, and the overall recall of the prediction was 0.45.

**Table 1:** Results of retrospective tests for the prediction of amino acid substitutions.

Test Year	The number of predicted substitutions	The number of actual substitutions	The number of correctly predicted substitutions	Recall	Precision
1998	9	8	8	1.00	0.89
1999	4	3	1	0.33	0.25
2000	4	5	2	0.40	0.50
2001	10	5	5	1.00	0.50
2002	7	3	1	0.33	0.14
2003	13	12	11	0.92	0.85
2004	4	5	3	0.60	0.75
2005	2	0	0	-	0.00
2006	4	2	2	1.00	0.50
2007	5	2	0	0.00	0.00
2008	4	1	0	0.00	0.00
2009	7	1	0	0.00	0.00
overall	73	47	33	0.70	0.45

Recall was defined as the number of correctly predicted substitutions divided by the total number of actual substitutions. Precision was defined as the number of correctly predicted substitutions divided by the total number of predicted amino acid substitutions.

To assess the validity of the result of the retrospective tests, we repeated similar retrospective tests with other methods and compared the results (Table 2). First we tested a method that randomly selects an HA sequence for each year. With 100 sets of tests, overall precision and recall were 0.24±0.015 and 0.22±0.012 respectively, showing low predictive ability as expected. Secondly we tested method that selects the HA sequence that has the maximum numbers of substitutions at the 18 positively selected codons identified by Bush et al [10]. Although the overall precision and recall were much higher than random tests, the accuracy of prediction was lower than that of our method. Three methods that select the sequence that has the maximum number of amino acid substitutions from current and past dominant sequences yielded higher recalls. However, the overall precision was lower than the method using 18 positively selected codons. Among all the methods we tested, the gamma-distribution-based method was the only method that yields higher recall and higher precision than Bush's methods.

**Table 2:** Comparison of overall recall and precision with other methods.

Method	Overall Recall	Overall Precision
Select a sequence randomly (n = 100)	0.22±0.012	0.24±0.015
Select the one that has the maximum numbers of substitutions at the 18 codons identified by Bush et al	0.49	0.34
Select the one that has the maximum numbers of substitutions from the current dominant sequence	0.51	0.13
Select the one that has the maximum numbers of substitutions from past dominant sequences	0.55	0.25
Select the one that has the maximum numbers of substitutions from the dominant sequence of two years ago	0.70	0.23
Select the one that has the maximum numbers of substitutions at antigenic domains A–E	0.70	0.32
Select the one that has the minimum errors from the gamma-distribution-based expectation	0.70	0.45

## Discussion

Our study found that the long-term evolution of HA was reasonably characterised by a ‘bonsai-like’ pathway of which trunk was constantly curved in the MDS-constructed 3D space. This unique property of the sequence evolution indicated that the evolution on the trunk favoured multiple substitutions at the same positions on HA molecules. Our study found that the curvature was relatively constant and reasonably explained by assuming that the rate of amino acid substitutions on HA varied from one position to another according to a gamma distribution. The estimated parameters of the gamma distribution allowed us to predict the amino acid substitutions on HA in subsequent years with reasonable accuracy.

The small value of its gamma parameter estimated in this study suggested that most of amino acids on HA remained unchanged, but amino acid substitutions occurred at a relatively restricted number of positions on the HA. The result was consistent with previous studies identifying several positions that had undergone the positive Darwinian selection, where non-synonymous mutations have been favoured [16], [17], [28], [31], [32]. Although the positions that undergo amino acid substitutions could have been moving to different positions over time [33], our analysis indicated that the relative sequence distance between two trunk sequences remained roughly constant with respect to the difference in their isolation years. This stable feature allowed us to predict the relative sequence distance between two viruses located on the trunk, and led us a fully-computerized prediction method.

He and Deem have recently pointed out that an MDS visualization with density estimation allowed us to identify a cluster of ‘incipient dominant strains’ before it became dominant [15]. They proposed two important criteria for the selection of a new vaccine strain. The first criterion is that a new cluster that does not contain currently circulating strains or vaccine strains is detected. The second criterion is that the current vaccine strain does not provide high protection against strains in the new cluster. The reason why our gamma-distribution-based model achieves high recall and precision can be explained by their two criteria. First of all, the recognition of a Leading Bud conceptually corresponds to the detection of a newly emerging cluster of incipient dominant strains. Since a Leading Bud described in this paper should have a certain amount of different amino acids from dominantly circulating strains, a Leading Bud can be considered as one of the early isolates in a newly emerging cluster. Although we do not consider whether Leading Buds form a cluster or not, this property partially fits their first criterion. Secondly, our gamma distribution-based method recognizes a Leading Bud by finding an HA sequence that has the amino acid substitutions at the same positions as those seen in the past evolution. Since most of past amino acid substitutions are concentrated in the antigenic sites (Table S1), it is highly likely that the Leading Bud having amino acid substitutions at these positions antigenically differ from the dominantly circulating strain and the vaccine strain. This could meet the second criterion.

The overall recall of our prediction method was around 0.70, indicating that the model had a reasonable ability to predict amino acid substitution in the subsequent year for each year. It should be noted that this high recall was achieved by the prediction method that relied only upon the number of different positions in the amino acid sequences and the isolation year of the viruses. The overall precision, on the other hand, was around 0.45. Some of mistaken predictions might be attributable to the delayed appearance of amino acid substitutions in the dominant sequences on the trunk. For instance, the predicted substitutions for I144N in 1999, R50G in 2000 and 2001, E83K V202I, W222R G225D in 2001, and V226I and S227P in 2002 did not occur in the next years, but rather 2 or 3 years later (Table S2). For these idle periods, the prediction method could have looked too far ahead, and the Leading Buds might be too early to be used as vaccine strains.

In the retrospective tests, we evaluated the prediction methods by comparing the amino acid sequences of the Leading Buds with the dominant sequences in subsequent years. It was also confirmed that WHO-recommended vaccine strains had amino acid residues that were identical to those predicted by our method (Table S2). The major difference lies in the timing. We accept that an overhasty selection of vaccine strains might lead antigenic mismatch between vaccine and epidemic strains. The prediction of the exact timing of the antigenic change could become a subject of future study. In addition to the timing of antigenic change, a careful investigation on their characteristics such as antigenicity and growth in embryonated hen eggs must be practically important for the vaccine selection.

Vaccine strains must be selected in order to match the antigenicity of viruses that will circulate in the influenza season. The antigenic cartography, which was developed by Smith et.al, enables us to accurately predict antigenic similarity between two virus strains based on a large collection of hemagglutination inhibition (HI) assay data [5]. In the period from 2005 to 2006, for instance, the circulating H3N2 viruses changed from A/California/7/2004-like viruses to A/Wisconsin/67/2005-like viruses [15], [34]. The Leading Bud found in 2005 was A/Okinawa/18/2005. The antigenic-cartography-based antigenic distance between A/Okinawa/18/2005 and A/Wisconsin/67/2005 was found to correspond to a twofold difference in HI titers of antisera [34]. Therefore, it is likely that the antigenicity of A/Okinawa/18/2005 could match the epidemic strain in 2006. Although the Leading Buds may not be perfect candidates for the vaccine strains, we believe that our prediction method could provide useful information for the formulation of influenza vaccines.

Retrospective tests for 2006–2007, 2007–2008, and 2008–2009 failed to predict the actual amino acid substitutions. The low recall and precision are likely due to the limited number of amino acid substitutions during these periods. Since antigenic changes of H3N2 viruses occur every 3–5 years in a punctuated manner [5], the conservation of dominant sequences in a few contiguous years is common in the evolution of H3N2 viruses. We have not taken such periodicity into account in our evolutionary model, and our method could not predict the exact timing when a dominant strain is replaced by another strain. This result highlighted the need to develop a method that can predict the exact timing of the antigenic change of the virus. However, the HA of the dominant H3N2 virus after the September in 2009 possessed 3 of 6 amino acid substitutions that were predicted by our method using the sequence data before August 2009 (Table S2). A/Perth/16/2009, a similar strain to our Leading Bud (A/Thailand/CU-B110/2009), was recommended as a vaccine strain for H3N2 viruses by WHO on Dec 1st in 2010.

Further understanding of the gnarled trunk might be achieved by combined efforts with experimental studies. Future research direction include the association of the gnarled trunk evolution with the prediction of antigenic evolution [5], [35], [36], the effect of mutations upon biological activity of the protein [37], [38], and the effect of cross immunity to previously circulating dominant strains [21], [22]. Finally, the 3D visualization technique we present here enables us to represent the direction of sequence evolution as well as sequence phylogeny, providing additional information that is not obtained via traditional phylogenetic analysis.

## Materials and Methods

### Sequence Data

Nucleotide sequences for HA genes of H3N2 influenza A viruses isolated from humans during the period from 1968 to 2009 were downloaded from the Influenza Virus Resource at the National Center for Biotechnology Information (NCBI) [39] on Feb 23 in 2010. The isolation date of the latest sequence was July 11^th^ 2009. After eliminating sequences that contained ambiguous nucleotide codes, 6,806 amino acid sequences of the HA1 domain were determined by translating the nucleotide sequences using the standard genetic code. All the amino acid sequences were 328 amino acids long. Nucleotide sequences that gave an identical amino acid sequence were grouped together, and the nucleotide sequence having the fewest mutations from the pandemic strain in 1968 was used as a representative. By removing all identical amino acid sequences except one, 2,640 unique amino acid sequences were obtained.

### MDS analysis

For every pair of the 2,640 amino acid sequences of the HA1 domain, the sequence dissimilarity, which is the total number of positions where the two sequences possess different amino acids, was calculated. The resulting 3,483,480 pair-wise dissimilarities were stored in a dissimilarity matrix. The SMACOF algorithm [24] was used to find the optimal coordination of all sequences in the 3D map to minimise the sum of squared errors:



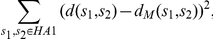
where 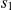
and 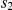
are sequences of the HA1 domain, 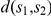 is the sequence dissimilarity, 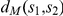
is the Euclidean distance in the 3D map.

### Amino Acid Substitution on the Trunk

A parsimony tree of HA was constructed from a total of 2,640 nucleotide sequences of the HA1 domain. The dnapars program in the PHYLIP package^24^ was used to construct the parsimony tree. The main trunk of the tree was defined as the longest path from the HA of the pandemic strain in 1968 to the HA of a strain circulating in 2009. The hypothetical nucleotide sequence on each trunk was translated into an amino acid sequence. For each residue position of the HA1 domain, amino acid substitutions found on the trunk were counted, and then the mean and variance of the substitution frequency were calculated and compared with a gamma distribution having the same mean and variance.

### Substitution Model

When assuming the variation of substitution rates follows a gamma distribution, the expected sequence distance between two sequences can be calculated by the formula 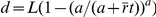, where 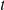 is the difference in their isolation years and 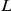 = 328 is the length of the HA1 domain. To obtain non-hypothetical amino acid sequences located near the trunk, a neighbour-joining tree was constructed from their nucleotide sequences. The tree construction was done using the dnadist and neighbor programs in the PHYLIP package [40] with the Jukes-Cantor distance option. For each trunk node of the neighbour joining tree, the amino acid sequence having shortest path to the trunk node was selected. Out of 2,640 HA sequences, 91 sequences were selected as trunk sequences (Table S3). For every pair of these selected sequences, their sequence dissimilarity (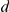) and the difference in isolation years (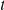) was recorded. By fitting the formula 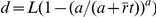
to the observed relationship between 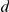
and 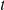, the gamma parameter (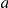) and mean substitution rate (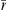) for our substitution model were estimated. In order to estimate the effect of the selection of trunk sequences on the Figure 3B, we performed a bootstrap resampling analysis. We made 100 datasets each containing 91 trunk sequences obtained by random resampling of the original 91 trunk sequences. Then, errors on the mean number of different amino acids were estimated using the 100 bootstrap datasets. As shown in the Figure S3, the errors of the means were estimated to be around one amino acid, suggesting that the result shown in Figure 3 has moderate robustness to the selection of trunk sequences.

### Dominant sequence

To define a representative sequence for each year, we adopted a strategy using the majority vote rule. For each year and each residue position, the dominant amino acid was determined as the amino acid that constituted the majority at the position in the year. The dominant sequence of a year was defined as the concatenation of the dominant amino acids of every position in the year.

### Prediction and retrospective tests

For each year, a Leading Bud, which is an amino acid sequence that will be located near the trunk in the next year, was predicted as follows. Let 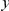 be a year. For a future HA sequence 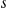
that would appear in the year 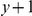, the expected number of different amino acids from each past trunk sequence 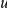
in year 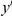 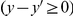
can be denoted by the formula 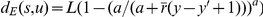. Thus, among sequences isolated in the year 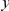, the sequence that is most likely to become a Leading Bud in the next year is formulated as the sequence 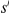, such that 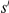 has the least sum of squared errors between sequence distance and expected distance from each trunk. The sum of squared errors is calculated by the formula:



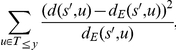
where 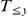 is a set of trunk sequences whose isolation years are earlier than or equal to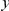. The amino acid sequence of the predicted Leading Bud was compared with the dominant sequence for the year, and a set of amino acid substitutions from the dominant sequence to the sequence of the Leading Bud was presented as predicted substitutions. After the prediction was made, the predicted substitutions were compared by the actual amino acid substitutions that occurred in the next year. Recall was calculated as the number of correctly predicted substitutions divided by the total number of actual substitutions. Precision was calculated as the number of correctly predicted substitutions divided by the total number of predicted amino acid substitutions.

## Supporting Information

Figure S1 **Scatter diagram of numbers of different amino acids vs. corresponding distances in the 3D map.** For every pair of two sequences, the actual numbers of different amino acids (X-axis) were plotted against corresponding distances in the 3D map (Y-axis). Horizontal bars show the mean values, and vertical lines indicate ±1SD of distances in the 3D map.
(EPS)

Figure S2 **Three-dimensional map of the human H1N1 influenza A viruses.** A total of 1228 amino acid sequences of the HA1 domain of human H1N1 influenza A viruses isolated during 1918 to 2010 are visualized in the 3D space. Each point represents an HA sequence, colour-coded by the isolation year of the virus. The whole coordination is determined by MDS analysis. All three axes represent sequence dissimilarity (spacing between grid lines represents 10 different amino acids), and the configuration can be freely rotated and translated. Shadows represent projections of points on a coordinate plane. Bold arrows on the left, middle, and right indicate a seasonal H1N1 virus isolated in 2009, the pandemic H1N1 virus in 1918, and the pandemic H1N1 virus in 2009, respectively.
(EPS)

Figure S3
**Boot strap resampling analysis of the mean substitution frequency on the trunk.** A total of 100 bootstrap datasets obtained were generated by random resampling of the original 91 trunk sequences. Errors on the mean number of different amino acids were estimated to be around one amino acid. A curve using the formula 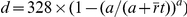 is superimposed (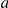 = 0.129, 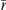 = 0.0118).(EPS)

Table S1 **The amino acid positions that were substituted one or more time.** The positions on HA where amino amino acid substitution were occurred on the trunk are shown with their frequency. Each alphabet represents the antigenic domain to which the position belongs.
(DOC)

Table S2 **Selected Leading Buds, predicted substitutions, and actual substitutions in the retrospective tests.** Correctly predicted substitutions are shown in bold-face. The predicted substitutions that did not occur in the next years but occurred 2 or 3 years later are underlined. Different amino acids on HA between a new WHO vaccine strain and preceding vaccine strain are shown in the rightmost column.
(DOC)

Table S3 **The HA sequences located near the main trunk.**
(DOC)

Movie S1 **Movie of 3D map of HA sequences of H3N2 human influenza A viruses.**
(MP4)

Movie S2 **Movie of 3D map of HA sequences of H1N1 human influenza A viruses.**
(MP4)
