# Supplementary material for: Gnarled-Trunk Evolutionary Model of Influenza A Virus Hemagglutinin
Source: PLoS One. 2011 Oct 10;6(10):e25953. doi: 10.1371/journal.pone.0025953 (PMC3189952; doi:10.1371/journal.pone.0025953)
Supplement: Table S1 — The amino acid positions that were substituted one or more time. The positions on HA where amino amino acid substitution were occurred on the trunk are shown with their frequency. Each alphabet represents the antigenic domain to which the position belongs. (DOC) [file pone.0025953.s004.doc]

Table S1. The amino acid positions that were substituted one or more time

| **The frequency of amino acid substitutions** | **Amino acid positions (Antigenic Site)** |
| --- | --- |
| 1 | 31, 53(C), 54(C), 57(E), 63(E), 75(E), 78(E), 82(E), 94(E), 122(A), 126(A), 128(B), 140(A), 142(A), 143(A), 146(A), 157(B), 160(B), 163(B), 186(B), 188(B), 190(B), 192(B), 196(B), 202, 207(D), 213(D), 222, 227(D), 244(D), 248(D), 25, 260(E), 275(C), 299(C), 307(C) |
| 2 | 2, 3, 62(E), 112, 121(D), 131(A), 135(A), 137(A), 138(A), 158(B), 159(B), 173(D), 194(B), 197(B), 217(D), 242(D), 262(E), 276(C), 278(C) |
| 3 | 50(C), 83(E), 124(A), 133(A), 155(B), 172(D) |
| 4 | 144(A), 156(B), 189(B), 225 |
| 5 | 145(A), 226(D) |
| 6 | - |
| 7 | - |
| 8 | 193(B) |
