# Supplementary material for: Gnarled-Trunk Evolutionary Model of Influenza A Virus Hemagglutinin
Source: PLoS One. 2011 Oct 10;6(10):e25953. doi: 10.1371/journal.pone.0025953 (PMC3189952; doi:10.1371/journal.pone.0025953)
Supplement: Table S2 — Selected Leading Buds, predicted substitutions, and actual substitutions in the retrospective tests. Correctly predicted substitutions are shown in bold-face. The predicted substitutions that did not occur in the next years but occurred 2 or 3 years later are underlined. Different amino acids on HA between a new WHO vaccine strain and preceding vaccine strain are shown in the rightmost column. (DOC) [file pone.0025953.s005.doc]

Table S2. Selected Leading Buds, predicted substitutions, and actual substitutions in the retrospective tests.

| Test Year | Selected Leading Bud | Predicted substitutions ^1)^ | Actual substitutions ^1)^ | WHO vaccine strain | Substitutions of WHO vaccine strains ^2)^ |
| --- | --- | --- | --- | --- | --- |
| 1998 | CY006243 A/New York/508/1997 | **K62E T121N G124S V144I K156Q E158K V196A** I236L **N276K** | **K62E T121N G124S V144I K156Q E158K V196A N276K** | A/Sydney/5/97 | L3I K62E T121N G124S D133N G142S V144I K156Q E158K V196A Y233H N276K |
| 1999 | AF533722 A/Tucuman/V425/1998 | G129E **Y137S** L194I V223I | R57Q **Y137S** D172E | A/Sydney/5/97 | - |
| 2000 | CY002368 A/New York/325/1999 | R109K I144N **T192I** **D271N** | G5V Q33H K92T **T192I** **D271N** | A/Moscow/10/99 | I3L R57Q Y137S S142R K160R I194L A196T H233Y |
| 2001 | CY008804 A/Canterbury/85/2000 | **V5G** **H33Q** R50G **T92K** **I144N** T167A S199P S247C **N271D** P273S | **V5G H33Q T92K I144N N271D** | A/Moscow/10/99 | - |
| 2002 | EU857032 A/Hong Kong/CUHK51431/2001 | S46F R50G E83K **S186G** V202I W222R G225D | A106V N144D **S186G** | A/Moscow/10/99 | - |
| 2003 | DQ114505 A/Stockholm/25/2002 | **L25I R50G H75Q E83K V106A A131T D144N H155T V202I W222R G225D** V226I S227P | **L25I R50G H75Q E83K V106A A131T D144N H155T** Q156H **V202I W222R G225D** | A/Moscow/10/99 | - |
| 2004 | EU501294 A/NARA/64/2003 | I25V **Y159F** **S189N** **S227P** | K145N **Y159F** **S189N** V226I **S227P** | A/Fujian/411/2002 | L25I R50G H75Q E83K A131T I144N H155T Q156H R160K D172E S186G T192I T196A V202I W222R G225D I226V |
| 2005 | EF566166 A/Christchurch/263/2004 | K83R D291G |  | A/California/7/2004 | A138S K145N Y159F D188N S189N A196T V226I S227P |
| 2006 | EU501748 A/Okinawa/18/2005 | **S193F** **D225N** I274F G275C | **S193F** **D225N** | A/Wisconsin/67/2005 | N122D G186V N188D S193F T196A V223I D225N |
| 2007 | EU502295 A/Nepal/NP-HC90997-ORIGINAL/2006 | Q1S N6I T128A R142G K173E | G50E K140I | A/Wisconsin/67/2005 |  |
| 2008 | CY022882 A/USA/AF1028/2007 | Q33R T48I D85G K207R | K173Q | A/Brisbane/10/2007 | G50E D122N S138A K140I V186G L194P I223V |
| 2009 | CY037799 A/Ohio/UR07-0035/2008 | L3F K83N T128I L157S Q173N K276N A304S | K158N | A/Brisbane/10/2007 | - |
| 2010 | GQ902809 A/Thailand/CU-B110/2009 | E62K K92Q N144K P162Q N189K R261Q | (data was not available yet) | A/Perth/16/2009 | E62K N144K K158N K173Q N189K P194L I214S |

^1)^ Correctly predicted substitutions are shown in bold-face. The predicted substitutions that did not occur in the next years but occurred 2 or 3 years later are underlined.

^2)^ Different amino acids on HA between a new WHO vaccine strain and preceding vaccine strain are shown.
