# Supplementary material for: Gnarled-Trunk Evolutionary Model of Influenza A Virus Hemagglutinin
Source: PLoS One. 2011 Oct 10;6(10):e25953. doi: 10.1371/journal.pone.0025953 (PMC3189952; doi:10.1371/journal.pone.0025953)
Supplement: Table S3 — The HA sequences located near the main trunk. (DOC) [file pone.0025953.s006.doc]

Table S3. The HA sequences located near the main trunk

| **Accession number** | **Strain name** |
| --- | --- |
| AB295605 | A/Aichi/2/1968(H3N2) |
| AF201874 | A/Hong_Kong/1/68(H3N2) |
| AY661038 | A/Bilthoven/15793/68(H3N2) |
| CY019907 | A/Albany/19/1968(H3N2) |
| CY021845 | A/Albany/10/1968(H3N2) |
| CY033521 | A/Hong_Kong/1-9-MA21-2/1968(H3N2) |
| CY034004 | A/Hong_Kong/1-11/1968(H3N2) |
| K03335 | A/England/878/1969(H3N2) |
| AY660996 | A/Bilthoven/6449/71(H3N2) |
| CY006683 | A/Hong_Kong/46/71(H3N2) |
| CY009356 | A/England/72(H3N2) |
| CY007971 | A/Guandong/243/72(H3N2) |
| AF201875 | A/England/42/72(H3N2) |
| CY003496 | A/Hong_Kong/14/74(H3N2) |
| AY661044 | A/Bilthoven/628/76(H3N2) |
| CY007611 | A/Memphis/19/78(H3N2) |
| CY020221 | A/Albany/14/1978(H3N2) |
| AF201843 | A/Thailand/1979 |
| AF201844 | A/Belgium/1981 |
| AY661025 | A/Netherlands/233/82(H3N2) |
| AF008868 | A/Oita/3/83(H3N2) |
| CY009068 | A/Memphis/2/85(H3N2) |
| AF008899 | A/Bangkok/(H3N2) |
| AF008881 | A/Kobe/768/88(H3N2) |
| AF386605 | A/Seoul/16/89(H3N2) |
| AY661062 | A/Singapore/36/89(H3N2) |
| AY661066 | A/Beijing/353/89(H3N2) |
| AF008686 | A/Shanghai/6/90(H3N2) |
| AF008659 | A/Kasauli/206/91(H3N2) |
| CY012760 | A/New_York/777/1993(H3N2) |
| AY661178 | A/Hongkong/56/94(H3N2) |
| AF386618 | A/Kwangju/1/97(H3N2) |
| AF386619 | A/Kwangju/4/97(H3N2) |
| EU856839 | A/Hong_Kong/CUHK12160/1997(H3N2) |
| CY006283 | A/New_York/517/1998(H3N2) |
| EU856826 | A/Hong_Kong/CUHK10632/1998(H3N2) |
| EU856829 | A/Hong_Kong/CUHK10954/1998(H3N2) |
| EF566078 | A/Christchurch/45/98(H3N2) |
| AF534039 | A/Neuquen/102/99(H3N2) |
| AF534041 | A/Santa_Fe/466/99(H3N2) |
| CY003632 | A/New_York/459/1999(H3N2) |
| EU856971 | A/Hong_Kong/CUHK31490/1999(H3N2) |
| CY000721 | A/New_York/177/1999(H3N2) |
| EU856953 | A/Hong_Kong/CUHK26907/2000(H3N2) |
| CY017339 | A/Western_Australia/7/2000(H3N2) |
| EU857018 | A/Hong_Kong/CUHK50080/2001(H3N2) |
| EU857032 | A/Hong_Kong/CUHK51431/2001(H3N2) |
| AY589653 | A/Cheonnam/432/2002(H3N2) |
| DQ114516 | A/Stockholm/27/02(H3N2) |
| EU501171 | A/SINGAPORE/2/2002(H3N2) |
| EU514626 | A/Wuhan/16/2002(H3N2) |
| EU856989 | A/Hong_Kong/CUHK34175/2002(H3N2) |
| EU501184 | A/TAIWAN/8/2002(H3N2) |
| CY006859 | A/New_York/214/2003(H3N2) |
| EU501221 | A/CHANTHABURI/219/2003(H3N2) |
| EU501280 | A/Mae_Hong_Son/330/2003(H3N2) |
| AY945274 | A/Nepal/1717/2004(H3N2) |
| EU501537 | A/SHIZUOKA/1/2004(H3N2) |
| CY026064 | A/Austria/139082/2004(H3N2) |
| EU501547 | A/SINGAPORE/38/2004(H3N2) |
| EU501437 | A/JIANGSU/FU137/2004(H3N2) |
| EU502305 | A/New_York/NY-04-1647/2004(H3N2) |
| CY015988 | A/Western_Australia/65/2005(H3N2) |
| EU501830 | A/WAKAYAMA/1/2005(H3N2) |
| EU502330 | A/Rhode_Island/RI-2005100001/2005(H3N2) |
| EU502184 | A/Florida/FL-JVT-15282/2005(H3N2) |
| EU502427 | A/Wyoming/WY-00120-ORIGINAL/2005(H3N2) |
| EU501633 | A/CHONGQING/652/2005(H3N2) |
| CY008404 | A/Canterbury/236/2005(H3N2) |
| EU103675 | A/Denmark/76/2006(H3N2) |
| EU103644 | A/Denmark/101/2006(H3N2) |
| EU221476 | A/Kenya/AF4041/2007(H3N2) |
| CY026019 | A/Illinois/UR06-0036/2007(H3N2) |
| EU516054 | A/Colorado/14/2007(H3N2) |
| CY031833 | A/Victoria/248/2007(H3N2) |
| CY030001 | A/Honduras/AF1101/2007(H3N2) |
| EU716459 | A/Texas/02/2008(H3N2) |
| CY040098 | A/Taiwan/70120/2008(H3N2) |
| CY030023 | A/USA/AF1109/2008(H3N2) |
| EU885501 | A/Florida/10/2008(H3N2) |
| CY032152 | A/Texas/AF1655/2008(H3N2) |
| EU779526 | A/Oklahoma/01/2008(H3N2) |
| CY032155 | A/Texas/AF1659/2008(H3N2) |
| CY032586 | A/Korea/AF1728/2008(H3N2) |
| CY037735 | A/Kentucky/UR07-0109/2008(H3N2) |
| CY036999 | A/Ohio/UR07-0140/2008(H3N2) |
| CY031926 | A/California/AF1403/2008(H3N2) |
| CY032575 | A/Arizona/AF1717/2008(H3N2) |
| FJ686925 | A/Tennessee/04/2008(H3N2) |
| EU852009 | A/Iowa/02/2008(H3N2) |
| CY034461 | A/Washington/AF1834/2008(H3N2) |
